# Trends in antiplatelet strategies 12-months following coronary stent placement in anticoagulated patients

**DOI:** 10.1186/s12872-023-03161-7

**Published:** 2023-03-08

**Authors:** Eleni M. Gamvroulas, Aubrey E. Jones, John A. Saunders, Tara L. Jones, Daniel M. Witt

**Affiliations:** 1 Huntsman Cancer Institute, University of Utah, UT Salt Lake City, USA; 2 Department of Pharmacotherapy, University of Utah College of Pharmacy, 30 South 2000 East, Room 4323, Salt Lake City, UT 84112 USA; 3 Department of Cardiology, University of Utah School of Medicine, Salt Lake City, UT USA

**Keywords:** Atrial fibrillation, Venous thromboembolism, Percutaneous coronary intervention, Drug eluting stent, Antiplatelet therapy

## Abstract

**Background:**

Antithrombotic guidelines for patients undergoing percutaneous coronary interventions (PCIs) and also requiring anticoagulant medications are evolving. This study describes changes to antithrombotic therapy and associated outcomes 12-months following PCI in patients requiring ongoing anticoagulation therapy.

**Methods:**

Records of patients identified from queries of electronic medical records were manually reviewed to verify changes to antithrombotic therapy from discharge to 12-months and at 12-months following PCI, and episodes of major bleeding, clinically relevant non-major bleeding (CRNMB), major adverse cardiovascular or neurological events (MACNE), and all-cause mortality outcomes during an additional 6-months follow-up.

**Results:**

Patients (*n* = 120) receiving anticoagulation therapy at 12-months post PCI were classified into the following groups according to antiplatelet therapy status: no antiplatelet therapy (*n* = 16), single antiplatelet therapy (SAPT) (*n* = 85), and dual antiplatelet therapy (DAPT) (*n* = 19). Between 12- and 18-months following PCI there were 2 major bleeds, 7 CRNMB, 6 MACNE, 2 venous thromboembolisms, and 5 deaths. All but one bleeding episode occurred in the SAPT group. The odds of remaining on DAPT at 12-months were higher in patients who had PCI for acute coronary syndrome (odds ratio [OR] 2.91, 95% confidence interval [CI] 0.96, 8.77), and in those experiencing MACNE in the 12-months following PCI (OR 1.95, 95% CI 0.67, 5.66), but these associations were not statistically significant.

**Conclusion:**

Most anticoagulated patients were continued on antiplatelet therapy 12-months post PCI. Bleeding was numerically more common in anticoagulated patients continuing SAPT therapy beyond 12 months. There was significant variability in antithrombotic prescribing patterns 12-months post PCI suggesting a potential opportunity for standardizing care in this patient population.

## Introduction

Atrial fibrillation (AF), venous thromboembolism (VTE), and percutaneous coronary interventions (PCI) require management with antithrombotic medications [1]. Prescribing appropriate therapy for patients receiving anticoagulant therapy for AF or VTE and antiplatelet therapy following PCI presents a challenge to clinicians as they attempt to balance the competing risks of thromboembolism reduction and increased bleeding risk associated with these medications. Evidence based prescribing guidelines pertaining to this situation have undergone substantial evolution over the past decade [2].

Commonly, patients with AF and additional stroke risk factors and patients with VTE are anticoagulated using either a direct oral anticoagulant (DOAC) or warfarin to reduce the risk of thromboembolic complications [1, 3]. PCI with implantation of bare metal or drug-eluting stents (DES) is an emergent intervention for patients experiencing acute coronary syndromes (ACS) such as myocardial infarction (MI) or unstable angina or an elective intervention for those with stable ischemic heart disease (SIHD) [4]. As acute stent thrombosis can lead to catastrophic MI, antiplatelet therapy with aspirin and/or P2Y_12_-inhibitors like clopidogrel, prasugrel, or ticagrelor is recommended during the 6-12-months following stent implantation [5, 6].

The two primary approaches for patients with AF or VTE who require PCI with DES placement are dual antithrombotic therapy and triple antithrombotic therapy. Dual antithrombotic therapy is the combination of oral anticoagulation therapy with single antiplatelet therapy (SAPT), typically a P2Y_12_-inhibitor [1]. Triple antithrombotic therapy is the combination of oral anticoagulation with dual antiplatelet therapy (DAPT), the combination of a P2Y_12_-inhibitor and aspirin [1]. Type and duration of antithrombotic therapy is determined based on the interplay between thrombotic risk factors and bleeding risk [1]. Dual antithrombotic therapy is preferred in low-risk patients requiring oral anticoagulation therapy (OAC) for AF or VTE [7]. Meta-analyses have consistently demonstrated less bleeding with dual vs. triple antithrombotic therapy and DOAC vs. warfarin-based antithrombotic therapy, but trials evaluating dual antithrombotic therapy have not been sufficiently powered to demonstrate effectiveness in preventing recurrent ischemic events [7].

The importance of balancing safety and effectiveness in antithrombotic regimens is the focus of ongoing research, but recent evidence-based guidelines provide recommendations regarding the initial 12-months of therapy following PCI in patients receiving oral anticoagulation therapy [7]. In general, these guidelines recommend limiting triple antithrombotic therapy to as short a time as possible, preferably 4 to 6 weeks [5–7]. Low risk of stent thrombosis or concerns regarding bleeding risk favor even shorter duration of triple therapy [7]. The extent to which these guideline recommendations have diffused into real-world clinical practice is unclear. Evidence-based recommendations for patients receiving anticoagulation for VTE who require PCI are not currently available.

Optimal antithrombotic therapy management beyond 12-months post PCI is less well understood. After 12 months, most drug-eluting stents pose less risk for thrombosis, reducing the need for antiplatelet therapy [8]. For patients who experience no additional coronary events during the year following PCI (i.e. SIHD), recent evidence-based guidelines recommend discontinuing antiplatelet therapy and continuing DOAC or warfarin monotherapy [6, 7]. Similar to guideline adherence during the initial 12 months post PCI, the extent to which recommendations pertaining to antithrombotic therapy beyond 12 months are followed in real-world clinical practice is unknown.

The objective of this study was to describe antithrombotic therapy prescribing patterns and clinical outcomes during two distinct time frames; the initial 12-months and between 12- and 18-months following PCI with DES placement in patients requiring ongoing anticoagulant therapy.

## Methods

### Study setting

This observational study was conducted using electronic data from the University of Utah Health System, the only academic health care system in the Mountain West. Electronic data were abstracted using a standardized form hosted in Research Electronic Data Capture (REDCap). REDCap is a web-based application that supports data capture in research studies. Study procedures were deemed exempt by our local Institutional Review Board (IRB_00085773) with a waiver of informed consent.

### Patient identification

**Fig. 1:**
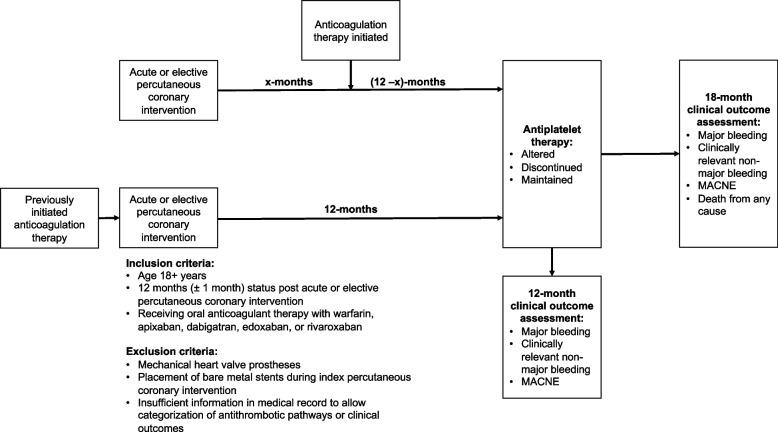
Overview of study patient identification, flow, and outcomes. MACNE- major adverse cardiovascular or neurological events

Patients were identified from queries of electronic medical records using International Classification of Disease 9th or 10th edition (ICD-9/10) and Current Procedural Terminology (CPT) codes for AF, VTE, and PCI. Electronic discharge records of hospitalized patients with ICD-10 codes in “any” diagnostic position for AF and/or VTE and CPT codes for PCI were manually reviewed to verify proper categorization of those receiving anticoagulation therapy to prevent stroke for AF or treatment of VTE who underwent PCI with placement of one or more drug-eluting stents. Adult patients (age 18 + years) who were 12 months (± 1 month) status post PCI and who were concurrently receiving oral anticoagulant (OAC) therapy with warfarin, apixaban, dabigatran, edoxaban, or rivaroxaban were considered for study inclusion. Patients initiating OAC therapy either before or after PCI were included providing that 12-months (± 1 month) had elapsed since the index PCI. Patients with mechanical heart valve prostheses and placement of bare metal stents during index PCI were excluded as were those with insufficient information in their medical record to allow categorization of antithrombotic pathways or clinical outcomes. An overview of the study design is provided in the Fig. 1. Baseline characteristics collected administratively or during manual medical record reviews included age, sex, race, ethnicity, comorbid diseases, reason for PCI (ACS/MI or SIHD), oral anticoagulant type, Charlson Comorbidity Index (CCI) and CHA_2_DS_2_-VASc scores (patients with AF).

### Outcomes

The primary outcomes of interest were the rates of various antithrombotic therapy prescribing patterns during the initial 12-months following PCI and changes (if any) to antithrombotic therapy at 12-months (± 1 month) following the index PCI. Changes were categorized according to the predominant antithrombotic therapy pathways identified during manual medical record reviews. After categorization of antithrombotic therapy changes at 12-months, patients were followed an additional 6-months for clinical outcomes including major bleeding, clinically relevant non-major bleeding (CRNMB), major adverse cardiovascular or neurological events (MACNE), and all-cause mortality.

Clinical outcomes were identified using hospitalization ICD-9/10 codes for bleeding and thromboembolic events in “any” diagnostic position and confirmed via manual chart review. Bleeding outcome severity was categorized using the definitions of the International Society on Thrombosis and Haemostasis (ISTH). Specifically major bleeding was defined as fatal bleeding, symptomatic bleeding in a critical area or organ, or bleeding that caused a drop in hemoglobin level of 2.0 g/L or more, or that lead to the transfusion of two or more units of whole blood or red blood cells [9]. CRNMB was defined as any bleeding that required medical intervention by a healthcare professional, lead to hospitalization or an increased level of care, or prompted a face-to-face evaluation but did not meet the definition of major bleeding [10]. MACNE was defined as the occurrence of cardiovascular death, MI, stroke/non-central nervous system systemic embolism or transient ischemic attack [11]. All-cause mortality was identified through documentation of death within the EMR.

### Statistical analysis

Baseline characteristics were summarized using proportions for categorical variables and means and standard deviations or median with interquartile range for continuous variables. The proportion of patients falling into the most common antithrombotic therapy pathways was calculated by dividing the number of patients in a given pathway by the total number of patients included in the analysis. The incidence rates of clinical outcomes were calculated by dividing the number of patients experiencing a given outcome during the applicable follow up period by the total number of patients in a given group and reported as a percentage. Differences in baseline characteristics between antithrombotic pathways were compared using analysis of variance (ANOVA), the chi-squared test of association or Fisher’s exact test as appropriate. Logistic regression models were used to examine the association between antithrombotic therapy pathway at 12-months and reason for initial PCI and the occurrence of MACNE between 0- and 12-months post-PCI. The relative risk [RR] of experiencing the composite outcome between 12- and 18-months post PCI for the various APT groups was calculated along with 95% confidence intervals [CI]. Data analysis were performed using Stata, v.17 (StataCorp LLC, College Station, TX). P-values < 0.05 were considered statistically significant.

## Results

From July 1, 2014 to July 1, 2018, 242 patients were identified from electronic medical record queries. Of these, 120 met inclusion criteria for the final analysis. Reasons for exclusion included insufficient information in the electronic medical record (*n* = 64), death within 12-months post PCI (*n* = 24), no OAC at 12-months post PCI (*n* = 34), bare metal stent (*n* = 1), and did not have PCI (*n* = 1). Some patients met more than one exclusion criteria. Relevant patient characteristics for the final cohort are summarized in Table 1. The mean age of patients was 67.4 years (standard deviation [SD] 13.3) and 71.7% were male. Patients were primarily of White race (90.8%) with non-Hispanic ethnicity (95.8%). Multiple comorbid conditions were present, including diabetes (45.8%), hypertension (83.3%), vascular disease (18.3%), and heart failure (27.5%). The median Charlson Comorbidity Index Score was 4 (interquartile range 3–7). The initial PCI type was elective for SIHD in 57.5% of cases.

**Table 1:** Baseline characteristics of patients requiring antithrombotic therapy following percutaneous coronary intervention (PCI) categorized by antiplatelet therapy status at 12-months post-PCI

	**All patients** ***n*** **=120, (%)**	**No APT,** *** n*** **=16 (%)**	**SAPT, ** ***n*** **=85 (%)**	**DAPT,** *** n*** **=19 (%)**
Mean Age (SD)	67.4 (13.3)	68.4 (17.9)	68.2 (12.6)	66.2 (12.6)
Male Sex*	86 (71.7)	11 (68.8)	65 (76.5)	10 (52.6)
Mean body mass index (SD)	31.8 (8.3)	28.3 (6.8)	33.2 (8.5)	30.1 (4.6)
Median serum creatinine (IQR)	1.1 (0.9, 1.4)	1.1 (1.0, 1.4)	1.1 (0.9, 1.3)	1.3 (0.9, 1.6)
Race & Ethnicity				
White	109 (90.8)	15 (93.8)	77 (90.6)	17 (89.5)
Black	5 (4.2)	0	5 (5.9)	0
Asian	1 (0.8)	1 (6.3)	0	0
Alaskan/Native American	1 (0.8)	0	1 (1.2)	0
Not specified	4 (3.3)	0	2 (2.4)	2 (10.5)
Hispanic ethnicity**	5 (4.2)	0	1 (1.2)	4 (21.1)
Smoking Status				
Never	59 (49.2)	10 (62.5)	41 (48.2)	8 (42.1)
Current	12 (10.0)	1 (6.3)	9 (10.6)	2 (10.5)
Former	42 (35.0)	5 (31.3)	30 (35.3)	7 (36.8)
Unknown	7 (5.8)	0	5 (5.9)	2 (10.5)
Comorbid Conditions				
Hypertension	100 (83.3)	11 (68.8)	71 (83.5)	18 (94.7)
Diabetes Mellitus	55 (45.8)	5 (31.3)	38 (44.7)	12 (63.2)
Heart Failure	33 (27.5)	4 (25)	23 (27.1)	6 (31.6)
Vascular Disease	22 (18.3)	1 (6.3)	18 (21.2)	3 (15.8)
Cancer	19 (15.8)	3 (18.6)	15 (17.7)	1 (5.3)
Stroke	12 (10.0)	0	11 (12.9)	1 (5.3)
Median CHA_2_DS_2_-VASc score (atrial fibrillation patients only) (IQR)	4 (3, 5)	4 (3,5)	4 (3,5)	4 (3.5, 5)
Median Charlson Comorbidity Index (IQR)	4 (3,7)	4 (2.5, 6)	5 (3,7)	5 (3.5,7)
Elective PCI for stable ischemic heart disease***	69 (57.5)	9 (56.3)	54 (63.5)	6 (31.6)
Reason for Anticoagulation^a^				
Atrial Fibrillation	90 (75.0)	14 (87.5)	62 (72.9)	14 (73.7)
Pulmonary Embolism	11 (9.2)	0	9 (10.6)	2 (10.5)
Deep Vein Thrombosis	10 (8.3)	0	8 (9.4)	2 (10.5)
Left Mural Thrombus	6 (5.0)	0	6 (7.1)	0
Hypercoagulable State	4 (3.3)	1 (6.3)	1 (1.2)	2 (10.5)
Other	8 (6.7)	2 (12.5)	6 (7.1)	0
Anticoagulation at time of PCI				
Warfarin	38 (59.4)	4 (50)	30 (62.7)	4 (57.1)
Apixaban	13 (20.3)	2 (25)	9 (18.4)	2 (28.6)
Rivaroxaban	10 (15.6)	2 (25)	7 (14.3)	1 (14.3)
Dabigatran	3 (4.7)	0	3 (6.1)	0
Anticoagulation initiated after PCI				
Warfarin†	41 (73.2)	3 (37.5)	27 (75.0)	11 (91.7)
Apixaban	7 (12.5)	2 (25.0)	4 (11.1)	1 (8.3)
Rivaroxaban	8 (14.3)	3 (37.5)	5 (13.9)	0

*APT* Antiplatelet therapy, *SAPT* Single antiplatelet therapy, *DAPT* Dual antiplatelet therapy, *SD* Standard deviation, *IQR* Interquartile range

^a^Patients could have more than one reason for anticoagulation

*p-value =0.05 for comparison between APT status groups

**p-value = 0.011 for comparison between APT status groups

†*p*-value=0.025 for comparison between APT status groups

The most common indication for anticoagulation was AF (75.0%) (Table 1). Patients anticoagulated prior to PCI comprised 53.3% of the cohort (*n* = 64), the remaining 46.7% (*n* = 56) initiated anticoagulation in the 12-months following PCI. Warfarin was the most commonly prescribed oral anticoagulant (59.4% and 73.2% of those who initiated anticoagulation before and after PCI, respectively).

Antiplatelet therapy prescribed after index PCI and changes to antiplatelet therapy during the ensuing 12-months are summarized in Table 2. Patients receiving anticoagulation therapy at 12-months post PCI were classified into the following groups according to antiplatelet therapy status: no APT (*n* = 16), SAPT (*n* = 85), or DAPT (*n* = 19). After PCI, most patients were discharged on dual antiplatelet therapy (83.3%). No patients received prasugrel. The most common antiplatelet regimens at discharge were aspirin 81 mg with clopidogrel (71.7%) followed by aspirin 81 mg with ticagrelor (10.8%) and clopidogrel alone (12.5%).

**Table 2:** Antiplatelet therapy prescribing patterns following percutaneous coronary intervention (PCI) categorized by antiplatelet therapy status at 12-months post-PCI

	**No APT,** ***n*=16 (%)**			**SAPT,** ***n*=85 (%)**			**DAPT,** ***n*=19 (%)**			**All patients** *n***=120, (%)**		
	**Post PCI**	**At 12 months**	**Post 12 months**	**Post PCI**	**At 12 months**	**Post 12 months**	**Post PCI**	**At 12 months**	**Post 12 months**	**Post PCI**	**At 12 months**	**Post 12 months**
**Time After PCI**												
ASA 81 mg alone	1 (6.3)	1 (6.3)	0	5 (5.9)	11 (12.9)	29 (34.1)	0	0	0	6 (5.0)	12 (10.0)	29 (24.2)
ASA 325 mg alone	0	0	0	0	1 (1.2)	1 (1.2)	0	0	0	0	1 (0.8)	1 (0.8)
Clopidogrel alone	3 (18.8)	11 (68.7)	0	12 (14.1)	61 (71.8)	51 (60.0)	0	0	0	15 (12.5)	72 (60.0)	51 (42.5)
Ticagrelor alone	0	1 (6.3)	0	0	4 (4.7)	4 (4.7)	0	0	0	0	5 (4.2)	4 (3.3)
ASA 81 mg + clopidogrel	10 (62.5)	0	0	60 (70.6)	6 (7.1)	0	16 (84.0)	17 (89.5)	17 (89.5)	86 (71.7)	23 (19.2)	17 (14.2)
ASA 81 + ticagrelor	2 (12.5)	0	0	8 (9.4)	1 (1.2)	0	3 (16.0)	2 (10.5)	2 (10.5)	13 (10.8)	3 (2.5)	2 (1.7)
ASA 325 + clopidogrel	0	0	0	1 (1.2)	0	0	0	0	0	1 (0.8)	0	0
Nothing	0	3 (18.8)	16 (100)	0	1 (1.2)	0	0	0	0	0	4 (3.3)	16 (13.3)
**Changes to antiplatelet therapy** ^a^												
	0-12 month	At 12 months		0-12 month	At 12 months		0-12 month	At 12 months		0-12 month	At 12 months	
No change	3 (18.8)	3 (18.8)		11 (12.9)	65 (76.5)		13 (68.4)	19 (100)		27 (22.5)	87 (72.5)	
Switched between APTs	2 (12.5)	0		6 (7.1)	11 (12.9)		1 (5.3)	0		9 (7.5)	11 (9.2)	
Discontinued APT	13 (81.3)	13 (81.3)		75 (88.2)	8 (9.4)		4 (21.1)	0		92 (76.7)	21 (17.5)	
Initiated a new APT	0	0		10 (11.8)	1 (1.2)		0	0		10 (8.3)	1 (0.8)	
Restarted a previously discontinued APT	0	0		2 (2.4)	1 (1.2)		4 (21.1)	0		6 (5.0)	1 (0.8)	

*APT* antiplatelet therapy, *SAPT* single APT, *DAPT* dual APT, *PCI* percutaneous coronary intervention, *ASA* aspirin

^a^More than one change in APT therapy was possible

During the 12-months following PCI, antiplatelet therapy changes included switching between antiplatelet agents (7.5%), discontinuing an antiplatelet agent (76.7%), and initiating a new antiplatelet agent (8.3%). Multiple changes in antiplatelet therapy were made in some patients. No change to antiplatelet therapy occurred in 22.5% of patients.

During the initial 12-months post PCI, there were 73 outcome events including MACNE (39, 32.5%), major bleeding (10, 8.3%), CRNMB (16, 13.3%), DVT (4, 3.3%), and PE (4, 3.3%) (Table 3). Of the MACNE events, 7 were strokes (17.9%). Bleeding sites included oral/nasal mucosa 9 (34.6%), gastrointestinal 6 (23.1%), genitourinary 5 (19.2%), skin 5 (19.2%), and retroperitoneal 1 (3.8%). The odds of remaining on DAPT at 12-months were nearly 3-fold higher in patients who had PCI for acute coronary syndrome (odds ratio [OR] 2.91, 95% confidence interval [CI] 0.96, 8.77), but this association was not statistically significant. The odds of remaining on DAPT at 12-months were also higher in patients experiencing MACNE in the 12-months following PCI, but this association was also not statistically significant (OR 1.95, 95% CI 0.67, 5.66).

**Table 3:** Clinical Outcomes following percutaneous coronary intervention (PCI) categorized by antiplatelet therapy status at 12-months post-PCI

	**No APT,** ***n*** **=16 (%)**		**SAPT,** ***n*** **=85 (%)**		**DAPT,** ***n*** **=19 (%)**		**All patients** ***n*** **=120, (%)**	
Time after PCI	0-12 month	12-18 months	0-12 month	12-18 months	0-12 month	12-18 months	0-12 month	12-18 months
Major Bleed	1, (6.3)	0	8 (9.4)	2 (2.4)	1 (5.3)	0	10 (8.3)	2 (1.7)
CRNM Bleed	1, (6.3)	1, (6.3)	13 (15.3)	6 (7.1)	2 (10.5)	0	16 (13.3)	7 (5.8)
MACNE	4 (25.0)	0	25 (29.4)	6 (7.1)	11 (57.9)	0	39 (32.5)	6 (5.0)
DVT	0	0	3 (3.5)	2 (2.4)	1 (5.3)	0	4 (3.3)	2 (1.7)
PE	0	0	3 (3.5)	0	1 (5.3)	0	4 (3.3)	0
All-cause Mortality	n/a	0	n/a	5 (5.9)	n/a	0	n/a	5 (4.1)

*PCI* Percutaneous coronary intervention, *APT* Antiplatelet, *CRNM* Clinically relevant non-major, *MACNE* Major adverse cardiovascular or neurological events, *DVT* Deep vein thrombosis, *PE* Pulmonary embolism

At 12 months following PCI, 13 (81.3%) patients in the No APT group discontinued remaining SAPT and 3 (18.8%) continued off all antiplatelet therapy. Patients in the No APT therapy group experienced one CRNM bleeding event between 12- and 18-months post PCI (6.3%) (Table 3).

In the SAPT group, 65 patients (76.5%) had no changes to the antiplatelet therapy they were receiving 12-months post PCI, 11 (12.9%) switched antiplatelet agents, 8 (9.4%) discontinued one DAPT antiplatelet agent, and 1 (1.2%) initiated a new antiplatelet agent. The majority of the SAPT group were receiving clopidogrel (60.0%), followed by aspirin 81 mg (34.1%) and ticagrelor (4.7%). Patients in the SAPT group experienced 2 major bleeds (2.4%), 6 CRNMBs (7.1%), 6 MACNE (7.1%), 2 DVTs (2.4%), and 5 deaths (5.9%) between 12- and 18-months post PCI (Table 3). Bleeding sites included 1 gastrointestinal (12.5%), 1 intracranial (12.5%), 2 skin (25%), 1 oral/nasal mucosa (12.5%), and 3 genitourinary (37.5%). There was one stroke (16.7%) in the patients experiencing MACNE.

Although some patients the DAPT group switched (5.3%) or discontinued/restarted (21.1%) antiplatelet therapy in the 12-months post PCI, all patients had no changes to the antiplatelet therapy regimen being taken at 12-months post PCI. A majority of patients stayed on aspirin 81 mg and clopidogrel (89.5%) with 10.5% remaining on aspirin 81 mg and ticagrelor. No patients in this group experienced adverse events between 12- and 18-months post PCI (Table 3).

Patients in the SAPT group were at numerically higher risk of experiencing the composite outcome between 12- and 18-months post-PCI than the no APT group (relative risk [RR] 3.20, 95% CI 0.46, 22.38) and the DAPT group (RR 8.14, 95% CI 0.51, 129.73), but these associations were not statistically significant.

## Discussion

We have described antiplatelet prescribing patterns in anticoagulated patients during the 18-months following PCI with a focus on changes, if any, occurring at 12-months. Our results showed that 86.7% of anticoagulated patients remained on at least one antiplatelet agent 12-months post PCI, with the most common being clopidogrel. All but one of the prespecified adverse clinical outcomes documented between 12- and 18-months post PCI occurred in patients who remained on antiplatelet therapy (all in the SAPT group), including 8 of the 9 recorded bleeding events. Evidence-based guidelines currently state that it is unnecessary to continue antiplatelet therapy beyond 12 months post-PCI in patients who have not experienced further coronary events [3, 5]. Our results show that this practice had not been widely adopted in our health system and that some patients were continuing both SAPT and DAPT longer than recommended in current guidelines. This observation suggests the potential need for evaluation of antiplatelet therapy prescribing in patients on anticoagulation therapy who have completed 12-months of therapy with an eye toward antiplatelet therapy deprescribing.

Prescribers in our study favored clopidogrel over other P2Y_12_-inhibitors. A recent network meta-analysis demonstrated similar risk of MACNE, major bleeding and death among different DAPT strategies following acute coronary syndrome patients, but did not include patients concurrently taking anticoagulation therapy [12]. Changes to antiplatelet therapy during the initial 12-month period following PCI were common in our study sample, illustrating the dynamic clinical course of patients undergoing PCI. Possible explanations for this observation include the impact of the higher cost associated with newer P2Y_12_-inhibitors and the fact that recent evidence has reinforced the importance of discontinuing triple antithrombotic therapy as soon as possible following PCI. This could in part explain why the most common antiplatelet therapy change during this timeframe was antiplatelet discontinuation. Adverse events were also common during the initial 12-months following PCI including MACNE and bleeding episodes. It is possible that these adverse events influenced antiplatelet prescribing decisions prior to and at 12-months following PCI. For example, patients who experienced MACNE prior to 12 months may have been more likely to resume previously discontinued aspirin and stay on triple therapy beyond 12 months due to perceived high risk for further thromboembolic complications. Indeed, we observed an almost 2-fold increase in the odds of remaining on DAPT at 12-months in patients who experienced MACNE in the 12-months following PCI, but this association was not statistically significant. Conversely, patients experiencing major bleeding or CRNMB may have been more likely to have stopped aspirin therapy prior to 12-months and to have discontinued clopidogrel at 12-months. Whether the index PCI was performed for ACS vs. SIHD could also have influenced antiplatelet therapy prescribing decisions, with those undergoing PCI for acute coronary syndrome being more likely to remain on antiplatelet therapy at 12-months. Patients remaining on DAPT at 12-months in our study were 3-times more likely to have undergone PCI for acute coronary syndrome, a result that approached statistical significance.

At 12-months post PCI, most patients continued the antiplatelet therapy they were taking at that time, whether SAPT or DAPT. Only 13 patients discontinued SAPT and only 7 patients discontinued DAPT. Of note, 11 patients in the SAPT group switched between antiplatelet agents but continued taking SAPT. In most cases this switch was from clopidogrel to aspirin. Unfortunately, we were unable to determine the clinical rationale for antiplatelet therapy decision making as this information was not consistently documented in the clinical narrative.

Our study covered a 4-year span between 2014 and 2018. It is possible that decisions to continue antiplatelet therapy in patients receiving OAC were consistent with evidence-based guidelines and prescribing norms during that time frame [13]. The 2020 European Society of Cardiology guidelines for managing AF recommend that 12-months after PCI for ACS, antiplatelet therapy should be stopped and OAC monotherapy continued (irrespective of the stent type) provided no recurrent ischemic events occurred in the interim [7]. However, this recommendation is based on a single open-label study comparing rivaroxaban monotherapy with combination therapy with rivaroxaban plus SAPT [14]. Although the study was stopped early due to increased mortality in the rivaroxaban plus SAPT group, the authors concluded that rivaroxaban monotherapy was noninferior to rivaroxaban plus SAPT for efficacy and superior for safety in patients with atrial fibrillation and stable coronary artery disease. This recommendation is not formally graded and is only found in the guideline text. The guideline was published in 2020, and the study upon which the recommendation was based was published in 2019. The 2020 European Society of Cardiology guidelines for the management of acute coronary syndromes in patients presenting without persistent ST-segment elevation make a graded recommendation to discontinue antiplatelet treatment in patients treated with anticoagulants after 12-months [6]. Many antiplatelet therapy prescribing decisions in our study were made prior to publication of these guidelines and it is therefore not surprising that most patients continued antiplatelet therapy 12-months following PCI. However, the aforementioned rivaroxaban study demonstrated superior safety (less bleeding) with a no APT strategy in patients taking oral anticoagulation therapy. It is possible that some of the 9 bleeding episodes observed in our study between 12 and 18 months post PCI could have been avoided had a no APT strategy been adopted.

There are limitations to our research including a relatively small sample size which resulted in imprecise estimates of study outcomes as evidenced by wide 95% CIs for the reported OR and RR estimates. Additionally, this was a single-center study and may not be generalizable to other healthcare systems. Also, as mentioned previously, we were not able to determine the clinical rationale underlying antiplatelet therapy prescribing decisions as this information was frequently missing from the clinical narrative. Additional evidence from adequately powered randomized, controlled, multicentered studies is needed regarding duration of antiplatelet therapy post PCI, thereby providing more information to a generalized population. Given that the prospective study discussed previously was stopped early due to excess mortality in the SAPT group, these types of studies may not be undertaken and evidence from high-quality observational studies may be the best that can be hoped for. Further, additional qualitative investigation could focus on factors that guided provider prescribing practices.

In conclusion, most patients in our study continued to use anticoagulation therapy in conjunction with OAC 12-months post PCI. Bleeding was numerically more common in anticoagulated patients continuing SAPT therapy beyond 12-months. This practice may not be aligned with recent data suggesting that antiplatelet therapy deprescribing should be considered in stable patients on anticoagulant therapy 12-months post PCI. Further, our results show significant variability in antiplatelet prescribing practices for anticoagulated patients throughout the 12-months post PCI and beyond. As OAC monotherapy appears to be associated with fewer bleeding complications without increased risk of secondary coronary events, APT deprescribing at 12-months post PCI should be considered for stable patients.

## Data Availability

The datasets during and/or analyzed during the current study may be available from the corresponding author on reasonable request.

## Abbreviations

ACS: Acute coronary syndrome
AF: Atrial fibrillation
ANOVA: Analysis of variance
CPT: Current procedural terminology
CRNMB: Clinically relevant non-major bleeding
DAPT: Dual antiplatelet therapy
DES: Drug eluting stent
DOAC: Direct oral anticoagulant
ICD: International classification of disease
ISTH: International Society on Thrombosis and Haemostasis
MACNE: Major adverse cardiovascular or neurological events
OAC: Oral anticoagulation therapy
PCI: Percutaneous coronary intervention
RR: Relative risk
SAPT: Single antiplatelet therapy
SIHD: Stable ischemic heart disease
VTE: Venous thromboembolism
